# Neuroimmune interactions: from molecular mechanisms to therapeutic targets

**DOI:** 10.1186/s43556-026-00565-7

**Published:** 2026-09-09

**Authors:** Yi-hang Hao, Rong-jia Shi, Ya-ling Tang, Xin-hua Liang

**Affiliations:** 1 State Key Laboratory of Oral Diseases & National Clinical Research Center for Oral Diseases & Department of Oral and Maxillofacial Surgery, West China Hospital of Stomatology, Sichuan University, Chengdu, China; 2 State Key Laboratory of Oral Diseases & National Clinical Research Center for Oral Diseases & Department of Oral Pathology, West China Hospital of Stomatology, Sichuan University, Chengdu, China

**Keywords:** Neuroimmune interactions, Neuroinflammation, Blood–brain barrier, Cytokines, Neurodegeneration, Molecular mechanisms

## Abstract

Neuroimmune interactions reveal that the central nervous system (CNS) is dynamically integrated with peripheral immunity. This bidirectional communication is mediated by microglia, astrocytes, peripheral immune cells, and the neurovascular unit through cytokines, chemokines, complement proteins, neurotransmitters, and neuropeptides. At the molecular level, pattern-recognition receptors, including Toll-like receptors and nucleotide-binding oligomerization domain-like receptors, activate NF-κB, MAPK, and JAK–STAT signaling. These pathways regulate cytokine production, oxidative stress, cellular metabolism, and glial phenotypes. NOD-like receptor thermal protein domain associated protein 3 (NLRP3) inflammasome activation induces caspase-1-dependent maturation of IL-1β and IL-18 and promotes pyroptosis, thereby amplifying neuroinflammation. Complement C1q/C3–CR3 signaling mediates synaptic pruning, whereas C–C motif chemokine ligand 2 (CCL2)–CCR2 signaling promotes leukocyte recruitment and microglial activation. Cytokines and matrix metalloproteinases disrupt endothelial tight junctions and compromise blood–brain barrier integrity. In addition, calcitonin gene-related peptide (CGRP) and substance P activate neuropeptide receptors to drive neurogenic inflammation. Together, these molecular circuits regulate glial activation, immune-cell trafficking, synaptic remodeling, neuronal excitability, vascular function, and cell survival. Their dysregulation contributes to neurodegenerative, neuroinflammatory, psychiatric, neurodevelopmental, and peripheral diseases through the blood–brain barrier, gut–brain axis, and vagus nerve. This Review summarizes how molecular neuroimmune mechanisms drive disease initiation, progression, and heterogeneity, and discusses emerging therapies targeting inflammasomes, complement, chemokine receptors, neuropeptide signaling, and microbiota to advance biomarker-guided, personalized neuroimmune medicine.

## Introduction

The nervous system and the immune system were long viewed as largely separate regulatory networks, one coordinating sensation, cognition and behavior, the other mediating host defence and tissue repair. This distinction has now been eroded by evidence that the two systems communicate continuously and bidirectionally through shared cells, soluble mediators and specialized anatomical interfaces, including the blood–brain barrier (BBB), meninges and peripheral ganglia [1–4].

We define neuroimmune interactions as this reciprocal crosstalk between neural and immune compartments, encompassing neuron–glia communication, trafficking of peripheral immune cells into the CNS, and signaling through cytokines, chemokines, complement, neurotransmitters and neuropeptides [5–7]. In health, these pathways support development, synaptic refinement, barrier integrity and immune surveillance; in disease, their dysregulation reshapes inflammatory tone and circuit function.

Accordingly, neuroimmune dysfunction is increasingly implicated in neurodegenerative, neuroinflammatory, psychiatric and neurodevelopmental disorders, as well as in peripheral conditions such as chronic pain, gut-brain-axis disorders and cancer [8–10]. While clinical successes of immunotherapies in multiple sclerosis (MS) and neuromyelitis optica have validated immune pathways as tractable therapeutic targets, such as Natalizumab [11], Anti-CD20 therapy [12, 13], Bruton’s tyrosine kinase inhibitors [14], the field remains fragmented across diseases and scales, and a unified framework linking mechanism to therapy is still lacking.

In this Review, we synthesize the cellular and molecular basis of neuroimmune communication, summarize its roles in central and peripheral disease, and highlight emerging therapeutic strategies, from precision biologics and small-molecule inhibitors to cell-based therapies, neuromodulation and microbiome-directed interventions [15–18]. Our aim is to provide a concise framework for translating neuroimmune biology into mechanism-guided treatment.

## Cellular architects of neuroimmune communication

### CNS-resident immune cells

CNS-resident immune cells, including microglia, astrocytes, and oligodendrocytes, constitute the intrinsic immune network of the brain and play essential roles in maintaining homeostasis, sensing injury, and shaping neuroinflammatory responses.

Microglia, yolk-sac-derived CNS macrophages, self-renew and continuously scan the parenchyma with motile processes, pruning synapses and clearing debris [5]. Threat detection triggers an amoeboid transformation and rapid transcriptional up-regulation of IL-1β, TNF-α, chemokines and ROS that, if sustained, fuels synaptic loss and neuronal death in disorders such as Alzheimer’s disease (AD). Single-cell profiling now resolves transcriptionally distinct sub-populations-disease-associated (DAM) and proliferative-region-associated (PAM) microglia-emphasizing context-dependent roles in neuroimmune homeostasis and pathology [6, 7]. Although activated microglia initially clear apoptotic neurons, their sustained release of TNF-α, IL-1β, chemokines and NOX2-derived ROS inflicts secondary damage on surviving neurons [19].

Astrocytes, the most numerous glial cells in the CNS, integrate homeostatic and inflammatory signals. They sense cytokines, chemokines and DAMPs via cognate receptors, and react with hypertrophy, GFAP up-regulation and context-dependent release of neurotrophic or pro-inflammatory mediators (IL-6, complement) [20, 21]. In MS and AD, reactive astrocytes form growth-inhibitory glial scars and disrupt synapses, while MAFG-driven subpopulations amplify inflammation. Targeting these specific astrocyte subsets is emerging as a precision therapeutic strategy [22].

Oligodendrocytes, once viewed solely as immune targets, express MHC-I and -II, present antigen to T cells, and secrete CCL2 after IFN-γ/TNF-α stimulation, actively shaping CNS immunity and recruiting leukocytes to lesions. By modulating the local inflammatory milieu, they regulate oligodendrocyte precursor cell differentiation and remyelination, thereby influencing functional recovery after immune-mediated demyelination [23, 24].

### Peripheral immune players

Although the CNS contains its own resident immune populations, peripheral immune cells contribute substantially to neuroimmune interactions, particularly under inflammatory or degenerative conditions. Among these, T lymphocytes are among the best characterized. In multiple sclerosis, autoreactive CD4⁺ and CD8⁺ T cells breach the BBB, recognize myelin antigens, and differentiate into Th1/Th17 effectors that secrete IFN-γ and IL-17, driving demyelination and axonal injury [8]. Clonally expanded CD8⁺ T cells in AD cerebrospinal fluid and IFN-γ-secreting T cells within aging neurogenic niches further highlight the detrimental impact of peripheral T-cell entry on neuronal survival and neurogenesis [9, 10].

B cells, traditionally viewed as antibody factories, now emerge as central pathogenic drivers in neuroimmune disorders through autoantibody production, antigen presentation to T cells, and secretion of pro-inflammatory cytokines. In MS, they form meningeal follicle-like structures that present myelin antigens to T cells and foster GM-CSF-secreting T-B synapses [25, 26], amplifying myeloid activation and BBB breakdown. Differentiation into plasma cells yields autoantibodies that activate complement and mediate tissue injury, as exemplified by aquaporin-4-specific IgG in neuromyelitis optica spectrum disorder (NMOSD). The clinical efficacy of anti-CD20 B-cell-depleting therapies in MS and related disorders provides definitive evidence for this B-cell-centric pathogenic axis [27].

Peripheral monocytes and their tissue-derived macrophage progeny constitute a distinct infiltrating population in CNS pathology. Under stress (injury, infection, chronic inflammation), CCR2⁺ Ly6C^hi monocytes breach the BBB [28], differentiate locally, and acquire context-dependent functions divergent from resident microglia: classical M1 macrophages amplify damage via TNF-α/IL-1β, whereas alternative M2 macrophages resolve inflammation through IL-10 and efferocytosis. In AD, infiltrating macrophages exacerbate cytokine milieu yet participate in amyloid-β clearance; single-cell secretion profiling further reveals that these macrophages suppress neuroprotective exosome release from neurons, while microglia enhance it [29]. Thus, therapeutic modulation must discriminate between infiltrating and resident myeloid subsets to preserve beneficial microglial functions while restraining macrophage-mediated injury.

### Neuronal regulation of immune cell function

Neuronal cells can regulate immune cell function through multiple mechanisms. First, neurotransmitters or neuropeptides released by neurons can modulate immune cell activation; for example, brain–lung signals transmitted by GABAergic neurons to ADRB2^+^ interstitial macrophages promote pulmonary inflammatory responses [30], and CGRP derived from nociceptors enhances dendritic cell function to drive autoreactive CD8^+^ T cell responses in vitiligo [31]. Second, neuron-derived signals can influence the production of inflammatory mediators by immune cells. Zhou et al. reported in a mouse model of hepatic ischemia–reperfusion injury that acidic neural signals suppress CCR2^+^ macrophage recruitment and the release of inflammatory mediators, thereby alleviating liver injury [32]. In addition, neurons can also affect the migration and localization of immune cells; at sites of skin and muscle injury, neurons send signals to immune cells to inhibit their recruitment, thereby promoting wound healing [33]. These examples illustrate how neural regulation of immune cells operates within the broader neurovascular context.

### The neurovascular unit and blood–brain barrier

#### Endothelial cells and BBB integrity

The neurovascular unit (NVU)—a complex of endothelial cells, pericytes, astrocytes and neurons—forms the anatomical and functional basis of the BBB [34]. Tight-junction-linked endothelium restricts paracellular diffusion, expresses transporters and receptors that gate nutrient and hormone flux, and actively regulates immune entry. Pro-inflammatory cytokines from microglia or infiltrating leukocytes up-regulate endothelial VCAM-1 and ICAM-1 [35, 36], capture circulating T cells and monocytes, and guide their transmigration into the parenchyma. In neurological disease, BBB "leakage" is both consequence and driver of pathology, allowing toxic solutes and excess immune cells to amplify neuroinflammation.

#### Pericytes and their immune functions

Pericytes, mural cells ensheathing capillary endothelium, are indispensable for BBB integrity. Beyond structural support, they actively sense inflammatory cues and secrete cytokines/chemokines e.g., CCL2 (MCP-1)—that chemoattract monocytes and T cells into the CNS [37]. Expressing MHC class II molecules, pericytes can present antigen to T lymphocytes, positioning them as local modulators of cerebral immunity [38]. In AD, pericyte loss compromises the BBB and promotes peripheral leukocyte infiltration [39]. Thus, pericytes are emerging as a critical yet under-appreciated hub linking BBB maintenance, immune-cell trafficking, and parenchymal immune regulation.

#### The role of the BBB in regulating immune cell trafficking

The BBB gates immune-cell entry via a four-step molecular cascade—selectin-mediated rolling, chemokine-triggered activation, integrin-dependent firm adhesion, and paracellular or transcellular diapedesis—that is constitutively repressed under physiological conditions but selectively amplified during neuroinflammation [40, 41]. Circulating T cells first engage E-/P-selectin to sample glycocalyx-anchored CCL2 or CXCL12; CCR2 or CXCR4 ligation initiates inside-out signaling that locks LFA-1 and VLA-4 onto endothelial ICAM-1/VCAM-1 [42], enabling diapedesis through tight-junction gaps or the endothelial body into the perivascular space and CNS parenchyma. Blockade of CCL2-CCR2 or CXCL12-CXCR4 signaling attenuates leukocyte trafficking in pre-clinical models of MS and neurodegeneration, validating the cascade as a therapeutically tractable axis.

## Molecular mechanisms of neuroimmune interactions

The nervous and immune systems communicate bidirectionally through a shared set of molecular mediators, including numerous common ligands and receptors. Pro-inflammatory cytokines not only mediate immune responses but also function as neuromodulators, influencing neurotransmitter release, synaptic plasticity, and neuroendocrine functions. The nervous system precisely modulates immune responses primarily through the secretion of neuropeptides and neurotransmitters. The immune system influences neural processes via multiple pathways. Peripheral immune cells and brain-resident immune effector cells secrete cytokines that play a central role in neuroimmune crosstalk [43]. Moreover, classical neurotransmitters can be synthesized by immune cells and can act on these cells through their cognate receptors, triggering downstream signaling pathways that further shape immune responses. Collectively, these receptor–ligand interactions and intracellular signaling cascades provide the molecular mechanisms underlying the intricate and dynamic communication between the nervous and immune systems.

### Cytokines and chemokines

Pro-inflammatory cytokines, including IL-1β, IL-6 and TNF-α, drive neuroinflammation via JAK/STAT, NF-κB and NLRP3 inflammasome signaling [15, 16].

The IL-1β/NLRP3 inflammasome pathway is a central driver of innate immune activation in neuroimmune disorders [44]. Activation of the NLRP3 inflammasome promotes caspase-1–dependent processing of pro-IL-1β into its mature form, thereby amplifying local inflammatory responses within the central nervous system [45]. Myeloid IL-1β suppresses α₁-adrenergic signaling in TH⁺ neurons, reduces enterochromaffin-cell differentiation and serotonin release, and ameliorates colitis [17, 18]. In microglia and infiltrating myeloid cells, this pathway contributes to cytokine release, BBB dysfunction, synaptic alteration, and neuronal injury. Importantly, IL-1β does not act merely as a downstream effector molecule; it also reinforces feed-forward inflammatory loops that sustain glial activation and broaden immune responses [46]. As a result, the IL-1β/NLRP3 axis represents a mechanistically coherent target linking danger sensing, inflammasome activation, and tissue damage.

The CCL2/CCR2 chemokine pathway is a representative mechanism for immune cell trafficking into the central nervous system. CCL2, also known as MCP-1, is produced by activated astrocytes, microglia, endothelial cells, and injured neurons, where it mediates recruitment of CCR2-expressing monocytes and other inflammatory cells. This chemotactic axis is particularly important in conditions characterized by breakdown of CNS immune privilege and peripheral immune cell infiltration. B-cell-derived lymphotoxin-α impairs neuronal excitability in MS [47], while IL-4-reprogrammed microglia enhance neurite outgrowth and recruit CCR2⁺/IL-4Rα⁺/arginase⁺ myeloid cells via IL-1β and CCL2 [48]. Beyond cell recruitment, CCL2/CCR2 signaling can amplify local inflammation by promoting glial activation, enhancing cytokine production, and sustaining monocyte-derived macrophage accumulation in the nervous system. Nociceptor-derived CCL2-CCR2 signaling orchestrates dendritic-cell–driven skin inflammation and adaptive immunity [49]. In addition, CX3CL1-CX3CR1 signaling links Wnt/Dkk3-deficient neurons to the phagocytic action of microglia, inducing depressive and anxiety-like behaviors in Parkinson's disease [50, 51]. CCL3-CCR5 signaling sensitizes neurons and maintains chronic pain by recruiting immune cells [52]. The CCL17/CCL22-CCR4 signaling pathway regulates arthritis, neuropathic pain, and postoperative pain under specific circumstances [53]. This indicates that chemokines coordinate neuroimmune interactions under various pathological conditions.

### Neurotransmitters and neuropeptides

Classical neurotransmitters—dopamine, norepinephrine, serotonin, GABA, and glutamate—serve as a shared molecular currency of neuroimmune communication, directly tuning immune-cell function through ligand-specific receptors. A hypothalamic corticotropin-releasing-hormone neuronal projection from the paraventricular nucleus to pulmonary sympathetic fibres releases norepinephrine that engages β₂-adrenergic receptors and β-arrestin-2 on neutrophils, blunts NF-κB signaling, and protects mice from acute lung injury [54]. Conversely, severe pneumonia recruits central-amygdala GABAergic neurons that enhance sympathetic output to lung interstitial macrophages, amplifying β₂-AR-mediated inflammation and worsening pulmonary damage [30]. During sepsis, spinal-astrocytic necroptosis depletes GABAergic neurons, removes central inhibition, and unleashes sympathetic overdrive that precipitates myocardial injury [55]. These circuit-specific, context-dependent mechanisms highlight the precision of neural immune control and identify discrete therapeutic nodes at the neuroimmune interface.

Neuropeptides—Substance P (SP), vasoactive intestinal peptide (VIP), calcitonin gene-related peptide, enkephalin and galanin—operate as potent, context-dependent immune regulators. SP–NK1R signaling promotes mast-cell degranulation, leukocyte recruitment and IgE production, fuelling colitis and asthma [56]. Conversely, VIP (and PACAP) via VPAC1/2 receptors suppresses pro-IL-1β, TNF-α and IL-12 release from macrophages and dendritic cells, skewing responses toward type-2 immunity and restraining epithelial stem-cell over-proliferation through the IL-25–ILC2–IL-13 axis [57–59]. VIPR2 engagement on ILC3s additionally curbs IL-22 production [60–62]. After spinal-cord injury, galanin fosters early neutrophil recruitment and later M1 → M2 macrophage transition [63], while a dedicated Penk⁺ Treg subset releases enkephalin to activate delta-opioid receptors on MrgprD⁺ nociceptors, suppressing neurogenic skin inflammation and producing peripheral analgesia [64, 65].

CGRP exerts multifaceted immunomodulatory effects across tissues and disease contexts. Neuron-derived CGRP suppresses type-2 innate lymphoid cell (ILC2) responses in allergic inflammation, whereas enteric β-CGRP released upon immune challenge bidirectionally tunes ILC2 activity to maintain intestinal immune homeostasis [66, 67]. During helminth infection, IL-4/IL-13 signaling via IL-13RA1 on primary enteric sensory neurons induces CGRPβ and neuromedin-U release, which activates muscularis macrophages, enhances arginase-1 expression, recruits eosinophils, and boosts anti-parasitic immunity [68]. In vitro, CGRP potentiates IL-4-driven M2 macrophage polarization in a time- and dose-dependent manner through calmodulin, PKC, and PKA pathways [69]. After acute tissue injury, nociceptor-derived CGRP engages RAMP1 on neutrophils and monocytes, inhibiting leukocyte recruitment, accelerating apoptosis, promoting efferocytosis, and driving pro-repair macrophage polarization via thrombospondin-1-dependent mechanisms [33, 70]. In medullary thyroid carcinoma, CGRP-induced cAMP elevation and KLF2 up-regulation impair dendritic cell function and correlate with reduced tumor-infiltrating T-cell activity [71]. During acute viral infection, neuron-derived CGRP signals through RAMP3-CALCRL on T cells, promoting TH1 differentiation and IFN-γ production while suppressing TH2 commitment via the CREB-ATF3-STAT1 axis, thereby enhancing antiviral immunity [72]. In rosacea, CGRP acting via RAMP1 on γδ T cells promotes their recruitment and IL-17A production, linking neuronal activation to cutaneous inflammation [73].

### Damage- and Pathogen-Associated Molecular Patterns (DAMPs/PAMPs) and their receptors

DAMPs and PAMPs bridge the nervous and immune systems via TLR/NLR engagement. Nociceptor-up-regulated TLR4/7/8 triggers MyD88/NF-κB/MAPK cascades, releasing cytokines, chemokines and neuropeptides [74] and governs neutrophil polarity [75]. Aβ, HMGB1, S100 proteins and nucleic acids sustain microglial/astrocytic activation through TLR/NLRP3 signaling, disrupt the BBB and amplify CNS inflammation [76–78].

Mitochondrial DAMPs (mtDAMPs)—heme, cytochrome c, cardiolipin, ATP, mtDNA, TFAM, N-formyl peptides and TCA metabolites—ignite cGAS-STING, NLRP3 and TLR signaling, provoking cytokine/chemokine release that damages neurons and recruits leukocytes [79–81]. In sporadic Parkinson’s disease (PD), damaged mtDNA is packaged into extracellular vesicles bearing ribosomal protein S3; dual TLR9/4 activation heightens oxidative stress and neuronal death [82]. In Alzheimer’s microglia, oxidative stress oxidises mtDNA guanine to 8-oxoguanine; the resultant Z-DNA is sensed by ZBP1, which recruits RIPK1 to drive type-I IFN, TLR and NLRP3 responses [83]. Up-stream, TDP-43 mis-localisation in HIV infection [84] and HK2-ubiquitination-controlled VDAC1 oligomerisation in sepsis-associated encephalopathy [85] promote mtDNA release and NLRP3 activation, causing synaptic dysfunction and cognitive deficits. N-formyl peptides further sustain regional neuroinflammation and neurodegeneration in MS via formyl-peptide receptor-1 [86].

### The complement system

Beyond host defence, the complement system functions as a neuromodulatory network that refines synapses during development but drives neurodegeneration when chronically activated. C1q–C3 tagging of presynaptic terminals directs microglial phagocytosis [87–91]. C1q blockade attenuates Guillain–Barré syndrome [92], whereas C1q/C3-CR3 signaling in the central amygdala underlies visceral pain–related synaptic remodelling [93]. Hippocampal C3a-C3aR/C5a-C5aR activation during sleep deprivation suppresses adult neurogenesis and impairs spatial memory [94]. In NMOSD, aquaporin-4 autoantibodies trigger astrocytic C3 release, which via C3aR-GSK3β/β-catenin inhibits dendritic arborisation; C3-C3aR inhibition restores neuronal complexity and cognition [95–97]. Similarly, C5 blockade prevents complement-mediated neuromuscular-junction destruction in myasthenia gravis, preserving synaptic transmission [98].

## Neuroimmune interactions in central nervous system disorders

**Fig. 1:**
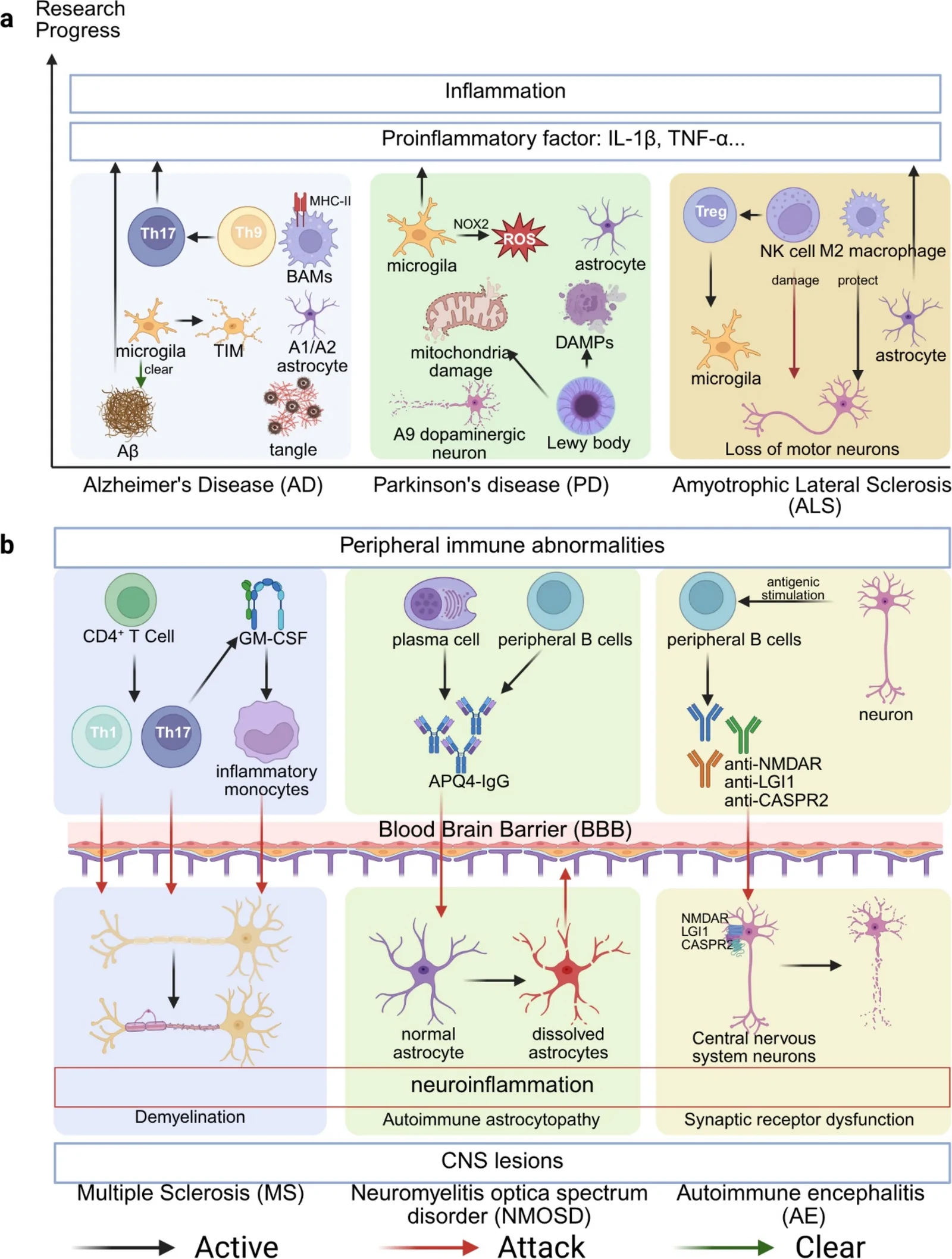
From protein aggregation to immune imbalance: an integrated map of neuroimmune interactions in central nervous system disorders. **a** The pathophysiological understanding of Alzheimer’s disease (AD) has shifted from the traditional amyloid-β (Aβ) plaque and tau tangle-centric paradigm to a neuroimmune-dominant framework characterized by terminal inflammatory microglia (TIM) generation and astrocytic polarization, accompanied by infiltration of peripheral immune cells—including Th9 and Th17 subsets—and expansion of MHC-II⁺ border-associated macrophages (BAMs). Parkinson’s disease (PD) is defined by the loss of A9 dopaminergic neurons in the substantia nigra pars compacta and the formation of Lewy bodies enriched in aggregated α-synuclein; these inclusions trigger mitochondrial dysfunction and act as damage-associated molecular patterns (DAMPs) to activate microglia, which subsequently release reactive oxygen species (ROS) and pro-inflammatory mediators alongside reactive astrogliosis. Amyotrophic lateral sclerosis (ALS) is marked by progressive degeneration of motor neurons, a process exacerbated by activated microglia and reactive astrocytes. Within the peripheral immune compartment, natural killer (NK) cells suppress regulatory T (Treg) cell recruitment, thereby sustaining microglial activation, whereas M2-polarized macrophages exert neuroprotection through secretion of interleukin-10 (IL-10) and neurotrophic factors. **b** Multiple Sclerosis (MS): Myelin-reactive Th1/Th17 cells produce GM-CSF and mobilize inflammatory monocytes and T cell-driven demyelination and glial inflammatory feedback; Neuromyelitis optica spectrum disorder (NMOSD): Peripheral B cells/plasma cells produce pathogenic AQP4-IgG which mediate autoimmune astrocytopathy; Autoimmune encephalitis (AE): Autoantibodies target neuronal surface or synaptic proteins, lead to autoantibody-induced synaptic receptor dysfunction; BBB breakdown permits entry of autoreactive lymphocytes, pathogenic antibodies and inflammatory mediators into the CNS. (Created with BioRender.com. BioRender agreement number: AOP0F6GHH0)

Neuroimmune interactions refer to the bidirectional communication between the nervous and immune systems through multiple cellular and molecular mechanisms. Recent studies have demonstrated that such interactions play a critical role in maintaining CNS physiology and in the pathogenesis of CNS diseases (Fig. 1).

### Neurodegenerative diseases

Neurodegenerative diseases are a group of neurological disorders characterized by progressive neuronal loss, including AD and PD. With the global aging population, these diseases have become major health challenges for the elderly [99]. Traditionally, neurodegenerative diseases were considered to result primarily from intrinsic neuronal dysfunction [100]. However, accumulating evidence indicates that neuroimmune and inflammatory responses may represent common pathophysiological mechanisms across multiple CNS disorders [101]. As a new paradigm in neurological research, neuroimmune interactions have achieved significant progress in recent years, offering important insights into the pathogenesis of neurodegenerative diseases and the development of novel therapeutic strategies.

#### Alzheimer’s disease

The pathological understanding of AD has shifted from the traditional focus on Aβ plaques and tau tangles [102] to a paradigm centered on neuroimmune responses as key drivers of disease. Aggregated Aβ maintains microglia in a primed, pro-inflammatory state; these cells release interleukin-1β (IL-1β), tumor necrosis factor-α (TNF-α), and reactive oxygen species (ROS), propagating a self-perpetuating inflammatory loop that exacerbates neuronal injury [103]. A functionally exhausted “terminal inflammatory microglia” (TIM) state—defined by CD11c⁺TMEM119⁺ cells with impaired phagocytic capacity—is markedly expanded in APOE-ε4 carriers and aged individuals, correlating with deficient Aβ clearance and accelerated pathology [104]. Genome-wide association studies consistently identify TREM2 and CR1 as risk loci that modulate disease progression by regulating microglial phagocytosis and metabolic fitness; loss-of-function mutations in TREM2 impair Aβ uptake and shift microglia toward a pro-inflammatory phenotype, thereby accelerating neurodegeneration.

Infiltration of peripheral immune cells further amplifies AD pathology. CD8⁺ effector-memory CD45RA⁺ (TEMRA) cells accumulate in the blood, negatively correlate with cognitive performance [8], and preferentially infiltrate brain regions enriched in tau pathology [105]. Three-dimensional human neuroimmune-axis models demonstrate that blockade of the CXCL10-CXCR3 or CCL3 axis partially inhibits T-cell recruitment [106, 107], thereby attenuating neuronal dysfunction [108]. CD4⁺ T-cell subsets exert distinct effects: Th1 cells promote Aβ clearance via interferon-γ (IFN-γ), whereas excessive activation exacerbates inflammation and cognitive deficits [9, 10]; Th17 cells, owing to their robust brain-infiltrating capacity, secrete interleukin-17A (IL-17A) and trigger neuroinflammation [109]; Th9 cells facilitate Th17 differentiation via IL-9 in synergy with transforming growth factor-β1 (TGF-β1) [110]. Border-associated macrophages (BAMs) residing at CNS boundaries exhibit age-related increases in major histocompatibility complex class II (MHC-II) expression and impaired extracellular-matrix degradation, contributing to vascular and glymphatic dysfunction and elevating AD risk [111]. Collectively, these findings indicate that strategies targeting microglial activation, chemokine signaling, and complement-mediated synaptic loss offer promising avenues for disease-modifying therapy in AD.

#### Parkinson’s disease

Parkinson’s disease (PD), the second most common neurodegenerative disorder, is defined by the selective loss of A9 dopaminergic neurons in the substantia nigra pars compacta and by the formation of Lewy bodies enriched in α-synuclein aggregates [112], resulting in a multitransmitter imbalance that includes striatal dopamine depletion. α-Synuclein drives pathogenesis through two complementary mechanisms: it precipitates mitochondrial dysfunction and oxidative stress [113], thereby disrupting neuronal ATP supply [114]; and it acts as a damage-associated molecular pattern (DAMP) that engages microglial TLR2/4/CD11b receptors and triggers NF-κB and MAPK inflammatory cascades [115].

Neuroimmune interactions in PD operate across several mechanistic layers. Among infiltrating peripheral immune cells, CD4⁺ T cells—particularly the pro-inflammatory Th17 subset—recognize α-synuclein epitopes and amplify the inflammatory milieu, whereas the contribution of CD8⁺ T cells remains to be fully characterized. Reactive astrocytes display impaired glutamate uptake, predisposing neurons to excitotoxic injury, and complement components co-localize with Lewy bodies, suggesting roles in pathological protein clearance or inter-neuronal propagation. Within the S100 family, S100A9 co-aggregates with α-synuclein under chronic neuroinflammatory conditions [116], whereas S100B exhibits a time-dependent expression profile—early elevation followed by normalization—and functions as an additional DAMP marker [117].

Emerging hypotheses and genetic evidence reinforce immune dysregulation as a central driver: gut–brain-axis studies propose α-synuclein pathology originating in the enteric plexus and ascending via the vagus nerve—truncal vagotomy correlating with reduced PD risk—while HLA-region polymorphisms link immune variability to susceptibility, collectively positioning microglial inhibition, peripheral immune blockade, and gut–brain immune interruption as promising therapeutic strategies.

#### Amyotrophic lateral sclerosis

Amyotrophic lateral sclerosis (ALS) is a fatal motor-neuron disease whose progression is propelled by persistent glial and peripheral immune activation; spinal microglia and astrocytes densely release TNF-α, IL-1β, glutamate and ROS, creating an excitotoxic-oxidative milieu that drives motor-neuron degeneration. Peripheral immunity contributes equally to this pathological cascade. Circulating and meningeal regulatory T cells (Treg) are reduced in number and suppressive function, permitting unrestrained expansion of cytotoxic CD8⁺ T cells that infiltrate the central nervous system through a disrupted blood–spinal cord barrier. These CD8⁺ T cells sustain motor-neuron major histocompatibility complex class I expression via interferon-γ (IFN-γ) and trigger neuronal apoptosis through Fas/FasL or granzyme pathways [118]. Natural killer (NK) cells accumulate in the motor cortex and spinal cord, where they recognize stress-induced NKG2D ligands on neurons and induce death through perforin release; concurrently, NK-cell-derived IFN-γ inhibits Treg recruitment and polarizes microglia toward an M1 pro-inflammatory phenotype, thereby amplifying the inflammatory loop [119, 120]. Conversely, monocytes that enter the central nervous system can differentiate into M2 macrophages, secrete interleukin-10 (IL-10) and neurotrophic factors, and confer partial protection to residual motor neurons [121].

### Neuroinflammatory and autoimmune diseases

The central nervous system (CNS) was long regarded as an “immune-privileged” organ; this concept has been definitively refuted. Contemporary neuroscience has demonstrated that the CNS harbors a distinctive immune ecosystem and engages in continuous, bidirectional communication with the peripheral immune system. These neuroimmune interactions are indispensable for CNS homeostasis, and their dysregulation represents a shared pathogenic foundation across multiple neuroinflammatory and autoimmune disorders.

#### Multiple sclerosis

Multiple sclerosis (MS) is a chronic inflammatory demyelinating disease of the central nervous system (CNS) that is initiated by aberrant peripheral adaptive immunity and subsequently amplified by intrinsic CNS immune cascades. The pathological sequence can be conceptualized as four inter-locking phases: peripheral sensitization, immune infiltration, glial positive feedback, and neurodegeneration.

Peripheral sensitization: in genetically susceptible individuals, environmental triggers polarize myelin-reactive CD4⁺ T cells toward Th1 (T-bet⁺/IFN-γ⁺) and Th17 (RORγt⁺/IL-17⁺) lineages [122] that highly express granulocyte–macrophage colony-stimulating factor (GM-CSF) [25, 26]. GM-CSF mobilizes Ly6C^hi classical monocytes from the bone marrow into the periphery, providing a cellular reservoir for subsequent CNS infiltration [28, 123].

Immune infiltration: once BBB integrity is compromised, Th1/Th17 cells, CD8⁺ tissue-resident memory T cells, and B cells sequentially enter the CNS parenchyma and meninges [27]. CD8⁺ T undergo clonal expansion within mixed active lesions and function as primary executors of compartmentalized autoimmune injury. B cells form ectopic meningeal follicle-like structures [124], secrete oligoclonal antibodies (OCB) and an array of pro-inflammatory cytokines (GM-CSF, TNF-α, IL-6, IL-12, IL-15), and potentiate T-cell activation via highly efficient antigen presentation, thereby sustaining the local inflammatory milieu.

Glial positive feedback: GM-CSF synergizes with IFN-γ to stimulate perivascular dendritic cells (cDC2) and macrophages [125], promoting continuous myelin antigen re-presentation and driving epitope spreading. Concurrently, GM-CSF polarizes microglia toward a pro-inflammatory M1 phenotype and induces astrocyte subpopulations to secrete additional GM-CSF [126], establishing a self-sustaining positive-feedback loop that amplifies inflammatory mediator release and exacerbates oligodendrocyte toxicity [127, 128].

Chronic inflammation drives oligodendrocyte death and demyelination, elevating serum neurofilament light chain as an axonal injury biomarker and allowing PET quantification of microglial activation for combined fluid-imaging surveillance. Immune homeostasis is maintained through negative regulation mediated by CD4⁺FoxP3⁺ regulatory T cells (Treg) and IL-10⁺ type-1 regulatory T cells (Tr1) [129]. EAE models demonstrate that Tregs suppress neuroinflammation and promote remyelination, whereas Tr1 cells resolve inflammation via IL-10. Another intranasal anti-CD3 trial, which safely induces Tr1 and IL-4, further supports this immunomodulatory approach for MS therapy.

#### Neuromyelitis optica spectrum disorder

Neuromyelitis optica spectrum disorder (NMOSD) is a recurrent, antibody-mediated autoimmune disease of the central nervous system that selectively targets optic nerves and spinal cord [130]. Unlike MS, NMOSD is initiated by aquaporin-4 (AQP4)-specific immunoglobulin G (AQP4-IgG). These autoantibodies enter the CNS at sites of BBB compromise, bind to AQP4 on astrocytic end-feet, and activate the complement cascade, culminating in the formation of membrane-attack complexes (MAC) that lyse astrocytes [131]. This “autoimmune astrocytopathy” produces near-complete loss of AQP4 immunoreactivity and further disrupts barrier integrity. Subsequent massive infiltration of neutrophils, eosinophils, and macrophages releases reactive oxygen species and proteases, provoking secondary demyelination and neuronal injury that define NMOSD lesions [132]. Monoclonal antibodies cloned from NMOSD patients recapitulate these histopathological features in ex vivo spinal-cord slices and in vivo rodent models, confirming the intrinsic pathogenicity of AQP4-IgG [133]. Mechanism-based blockade of the terminal complement pathway with eculizumab has significantly reduced relapse risk, establishing precision, antigen-specific therapy for NMOSD.

#### Autoimmune encephalitis

Autoimmune encephalitis (AE) comprises a spectrum of disorders in which autoantibodies directed against neuronal surface or synaptic proteins produce acute or subacute brain inflammation [134, 135]. Rather than mediating primary demyelination, these antibodies cause direct, antigen-specific neuronal dysfunction. Well-characterized molecular targets include the N-methyl-D-aspartate receptor (NMDAR), leucine-rich glioma-inactivated 1 (LGI1), and contactin-associated protein-like 2 (CASPR2). Pathogenic immunoglobulins are synthesized by peripheral B-cell clones and are readily detectable in serum and cerebrospinal fluid. Mechanistically, autoantibodies disrupt neurotransmission through several complementary pathways. Anti-NMDAR immunoglobulins cross-link and internalize receptors, decreasing synaptic NMDAR density and attenuating long-term potentiation [136]. Antibody–antigen engagement also elicits a secondary inflammatory cascade composed of CD8⁺ T-cell infiltration, microglial activation, and complement deposition, which collectively exacerbate psychiatric symptoms, seizures, and cognitive deficits. Early, antibody-guided immunotherapy—corticosteroids, intravenous immunoglobulin, or B-cell-depleting agents such as rituximab—substantially improves neurological outcome [137], underscoring the importance of prompt autoantibody identification.

AE exemplifies a unified principle: neuroinflammation and autoimmunity converge through continuous neuroimmune cross-talk. Peripheral loss of self-tolerance initiates adaptive-effector-driven autoimmunity, whereas dysregulated CNS-innate immunity triggers primary neuroinflammation; thereafter, innate activation amplifies adaptive cytotoxicity, and chronic intracerebral inflammation erodes tolerance to elicit de-novo autoantibodies (Table 1).

**Table 1:** Comparative neuroimmune mechanisms and therapeutic targets in CNS autoimmune and neuroinflammatory diseases

Feature	Multiple Sclerosis (MS)	Neuromyelitis Optica Spectrum Disorder (NMOSD)	Autoimmune Encephalitis (AE)
Primary Pathogenesis	Adaptive immunity-driven demyelination; Th1/Th17/GM-CSF⁺ T cells breach BBB [25, 26]	AQP4-IgG-mediated astrocytopathy with complement-dependent injury [131, 132]	Antibody-mediated neuronal surface antigen disruption; secondary T-cell inflammation
Key Immune Cells (Periphery)	Myelin-reactive CD4⁺ Th1/Th17 (GM-CSF⁺), B cells (ectopic follicles) [124, 131, 132]	AQP4-specific B cells/plasma cells [130]	B cells (autoantibody source); varying T-cell involvement
Key Immune Cells (CNS)	CD8⁺ T cells [129]; Microglia, astrocytes, oligodendrocytes [126]	Astrocytes (primary C1q/C3 target, MAC formation) [131]; neutrophils/eosinophils (secondary infiltration)	Neurons (direct antigen target); microglia (secondary activation)
Molecular Hallmarks	GM-CSF, IFN-γ, CCL2, IL-17, MMPs [123]	AQP4-IgG, C5b-9 MAC, GFAP elevation	Anti-NMDAR/LGI1/CASPR2 antibodies in serum/CSF, disrupted synaptic currents
BBB Integrity	Disrupted by Th17/MMP9; enables immune-cell diapedesis [27]	Early barrier vulnerability enables AQP4-IgG entry	Variable; often intact at onset
Main therapeutic strategy	T-cell trafficking blockade, B-cell depletion, GM-CSF/NLRP3 targeting [129]	Complement inhibition, B-cell depletion	Immunotherapy (steroids, IVIG, rituximab)
Key clinical challenge	Epitope spreading, progressive neurodegeneration	Accurate diagnosis and relapse prevention	Antibody heterogeneity and delayed recognition

### Psychiatric disorders

**Fig. 2:**
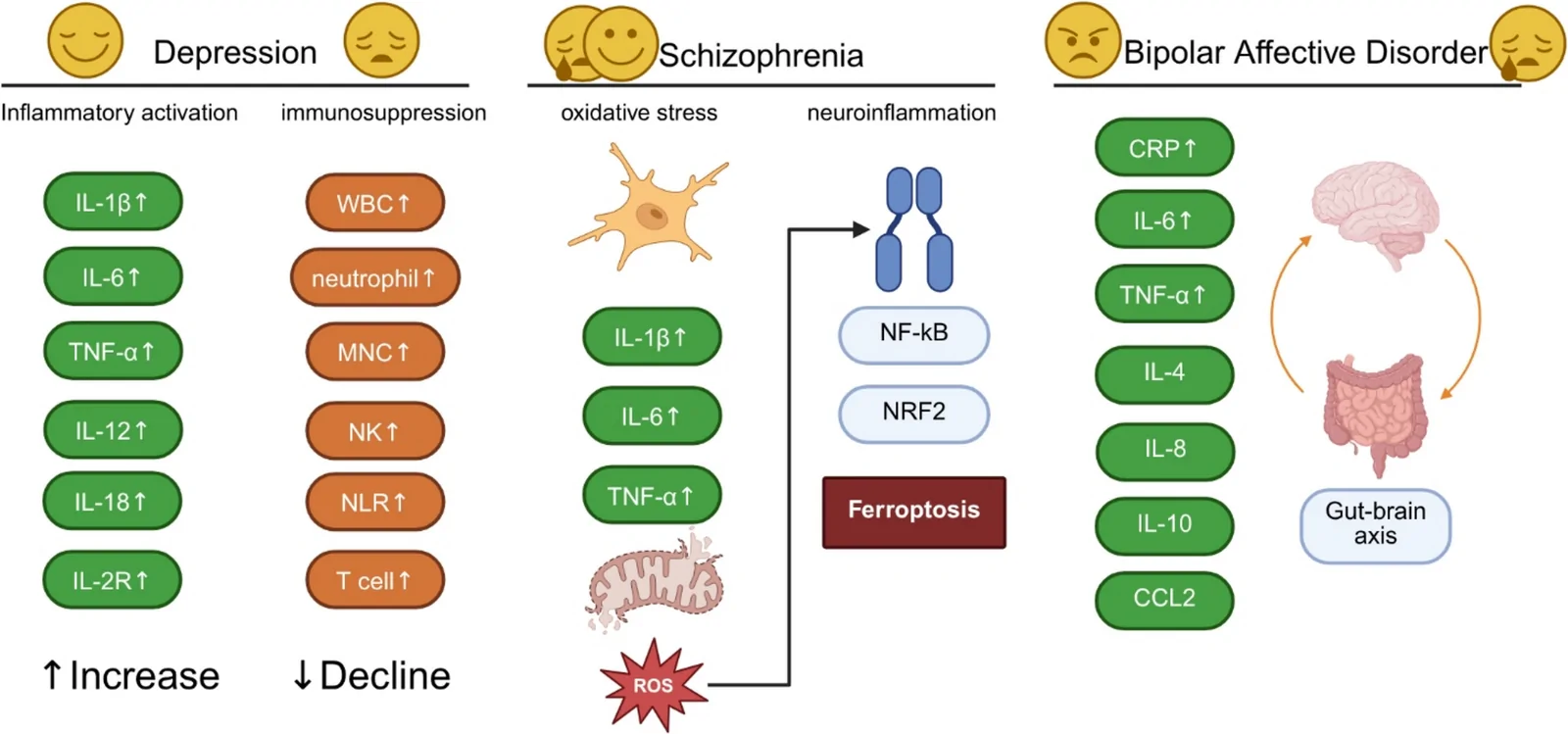
Neuroimmune regulation of mental disorders. Depression is characterised by simultaneous neuroinflammation and immune suppression: the former is driven by an array of pro-inflammatory mediators, whereas the latter involves multiple immune-cell subsets. In schizophrenia, oxidative stress and neuroinflammation coexist—oxidative stress arises from activated microglia releasing pro-inflammatory factors that impair mitochondrial function and increase reactive oxygen species (ROS), and neuroinflammation is subsequently triggered by ROS, engaging diverse inflammatory pathways (NF-κB, NRF2) and the ferroptotic cascade. Bipolar disorder features prominent pro-inflammatory mediator activation that is further amplified by gut–brain-axis dysregulation. (Created with BioRender.com. BioRender agreement number: REF033A50E)

The central nervous system (CNS) harbors a distinct immune ecosystem that engages in continuous, bidirectional communication with the peripheral immune system via cytokine gradients, meningeal lymphatics, and specialized vascular interfaces. This neuroimmune axis orchestrates neural development, refines synaptic plasticity, and coordinates post-injury tissue remodeling. Dysregulation of these signaling networks is increasingly recognized as a fundamental pathophysiological mechanism underlying a spectrum of psychiatric disorders, including major depressive disorder, schizophrenia, and neurodevelopmental syndromes (Fig. 2).

#### Depression

Major depressive disorder (MDD) is a prevalent and debilitating psychiatric illness. The neuroimmune hypothesis of MDD has transcended the “monoamine-deficiency” paradigm and is now redefined as a three-dimensional network disturbance encompassing “immune imbalance–neurotoxicity–metabolic stress” [138]. Consistent clinical and omics evidence shows that the acute phase of MDD presents a paradoxical state of “concomitant pro-inflammatory activation and immune suppression”: peripheral IL-1β, IL-6, TNF-α, IL-12, IL-18, and soluble IL-2 receptor are significantly elevated, positive acute-phase proteins (CRP, ceruloplasmin, α−1 acid glycoprotein) are increased, while negative proteins (albumin, transferrin) are decreased, indicating an IL-6/IL-1β-driven acute-phase response [139, 140]. Concurrently, reduced lymphocyte transformation, decreased NK-cell activity, and glucocorticoid resistance constitute an immune-suppressive phenotype that is causally intertwined with HPA-axis hyperactivation [141]. Bidirectional Mendelian randomization confirmed causal associations of IL-1β, IL-16, and CCL5 with MDD risk, whereas the reverse pathways were not significant [142].

At the immune-cell level, MDD patients exhibit increased absolute counts of peripheral leukocytes, neutrophils, monocytes, and NK cells, an elevated neutrophil-to-lymphocyte ratio (NLR), and baseline NLR predicts depressive episodes several years later [143, 144]; cerebrospinal fluid leukocyte counts are also significantly increased [145]. Transcriptomic analyses reveal enrichment of “neutrophil-mediated immunity” and “response to external biotic stimuli” pathways [146]; T-cell subsets display an activated phenotype, with elevated proportions of CD4⁺HLA-DR⁺, CD4⁺CD25⁺, CD4⁺CD71⁺, and CD4⁺CD40L⁺ cells, suggesting antigen-specific activation [147].

Pathways conveying inflammatory signals to the CNS include: cytokine passage across the BBB; vagal-solitary tract nucleus signaling; and gut dysbiosis–intestinal LPS translocation, which collectively induce microglial overactivation. Microglia up-regulate indoleamine 2,3-dioxygenase (IDO), shifting tryptophan metabolism from 5-HT synthesis toward the kynurenine pathway, depleting 5-HT and generating neurotoxic metabolites such as quinolinic acid that inhibit hippocampal neurogenesis and synaptic plasticity [148]. Recent studies further demonstrate that chronic stress induces peripheral immune cells to release matrix metalloproteinase 8 (MMP8), which directly modifies synaptic structures within reward circuits and precipitates depressive behaviors [149], providing a molecular node for “mind–body” mechanisms.

#### Schizophrenia

Schizophrenia is a severe, chronic psychotic disorder characterized by hallucinations, delusions, disorganized thought, and pervasive cognitive impairment. The dopamine hypothesis attributes positive symptoms to mesolimbic dopaminergic hyperactivity and negative/cognitive symptoms to mesocortical dopaminergic hypo-function [150]. Accumulating evidence indicates that neuroinflammation and oxidative stress form a self-reinforcing cycle that underlies these dopaminergic disturbances: microglial over-activation releases IL-1β, IL-6, and TNF-α, which stimulate NADPH oxidase and impair mitochondrial respiration, generating excessive reactive oxygen species (ROS) [151]. ROS, in turn, activate redox-sensitive transcription factors (NF-κB, NLRP3 inflammasome), perpetuating inflammation and oxidative damage.

Redox imbalance and mitochondrial dysfunction are central to schizophrenia-associated neurodegeneration [152]. Elevated ROS coincide with impaired antioxidant defenses (reduced glutathione, superoxide dismutase, catalase), resulting in lipid peroxidation, protein oxidation, and DNA damage that create a neuronal toxic micro-environment [153]. NF-κB and Nrf2 coordinately regulate this process: NF-κB amplifies pro-inflammatory signaling, whereas Nrf2-mediated antioxidant gene expression is suppressed, jointly exacerbating cellular injury [154]. Ferroptosis—GPX4-dependent, iron-catalyzed lipid peroxidation—has recently been implicated in progressive neuronal loss and cognitive deterioration in chronic schizophrenia [155].

Clinical data corroborate these mechanisms. Peripheral levels of malondialdehyde, 8-hydroxy-2'-deoxyguanosine, and nitrotyrosine are elevated, while total antioxidant capacity and glutathione peroxidase activity are reduced; both profiles correlate with symptom severity and cognitive decline. A 2025 multicenter study identified robust ferroptotic signatures—down-regulated GPX4, up-regulated ACSL4, decreased plasma glutathione, and altered iron metabolism—in chronic patients; these biomarkers significantly correlate with negative-symptom burden and cognitive dysfunction [156]. Oxidative stress preferentially targets parvalbumin-expressing fast-spiking interneurons, disrupting gamma-oscillatory networks that underlie working memory and sensory gating [157]. Collectively, these findings indicate that precision modulation of oxidative-stress and inflammatory cascades may offer novel therapeutic avenues for schizophrenia.

#### Bipolar disorder

Bipolar disorder (BD) is characterized by recurrent manic/hypomanic and depressive episodes that are sustained by dysregulated neuroimmune signaling. Meta-analytical data reveal a significant peripheral inflammatory signature: C-reactive protein (CRP) concentrations are elevated in both manic and euthymic patients, with the highest levels observed in drug-naïve cohorts, although absolute CRP values do not correlate with cross-sectional symptom severity [158]. Tumor necrosis factor-α (TNF-α) is consistently increased during acute episodes [159], whereas interleukin-6 (IL-6) displays state-dependent heterogeneity (elevation in mania, normalization in remission), limiting its utility as a phase-specific biomarker [160, 161]. Additional immune mediators including IL-4, IL-8, IL-10 and the chemokine CCL2 are quantitatively altered and contribute to disease pathogenesis.

These mediators converge on monoaminergic synapses, suppress adult hippocampal neurogenesis, and activate microglia changes mirrored in major depressive disorder. PET imaging, CSF soluble markers, gut dysbiosis and increased intestinal permeability collectively indicate gut–brain-axis dysfunction, motivating clinical evaluation of IL-6 or TNF-α blockade. Trans-diagnostic transcriptomes further reveal a shared immune-gene signature and conserved neuron–glial co-expression modules across bipolar disorder, schizophrenia and major depressive disorder [162], offering a data-driven roadmap for broad-spectrum immune-targeted therapy across mood and psychotic spectra.

### Neurodevelopmental disorders

Neurodevelopmental disorders (NDDs) arise from disruptions to early brain maturation. Although monogenic mutations remain prototypical aetiologies, converging evidence establishes that dysregulated neuroimmune interactions critically orchestrate developmental trajectories [163], and constitute an independent pathogenic substrate for polygenic NDDs.

#### Autism Spectrum Disorder (ASD)

Autism spectrum disorder (ASD) is a polygenic neurodevelopmental syndrome that exhibits a 4:1 male predominance; accumulating evidence indicates that sex-specific neuroimmune regulation underlies this dimorphism [164]. Placental deficiency of the neurosteroid allopregnanolone (ALLO) reduces microglial ramification and precipitates cerebellar hypomyelination, yielding autistic-like phenotypes exclusively in male offspring. Maternal immune activation (MIA) is a well-validated environmental risk factor for ASD [165]. Clinical cohorts and maternal-poly(I:C) mouse models demonstrate that MIA offspring display innate-immune over-activation that persists into adulthood; inappropriate initiation of fetal immune responses thus imprints long-term immune dysfunction. Peripheral immune dysregulation in children with ASD is multi-layered. (1) TLR4-activated monocytes secrete increased IL-1β, IL-6 and TNF-α [166]; (2) Chemokine profiles are aberrant: MCP-1, RANTES, IL-8, CXCL1 and MIP-1α/β are elevated, whereas IP-10/CXCL9 is reduced [167]; (3) Myeloid dendritic-cell (mDC) numbers are expanded and display high surface expression of CD80/CD86; these changes correlate with amygdala enlargement and social-communication deficits [168]; (4) Natural-killer (NK)-cell counts are increased and RNA-sequencing confirms selective activation of NK-cell cytotoxicity pathways [169], and its dysfunction contributes to ASD pathology. Long-term immune dysregulation may stem from epigenetic reprogramming-mediated immune training, leading to persistent myeloid cell activation or abnormal hematopoietic stem-cell differentiation. Such chronic immune abnormalities, together with microglial dysfunction, constitute core pathological links in ASD.

#### Role of maternal immune activation

Maternal immune activation (MIA) denotes a gestational systemic inflammatory response elicited by infectious or non-infectious stimuli and represents a core environmental risk factor for neurodevelopmental disorders (NDDs) including autism spectrum disorder and schizophrenia in offspring [170]. Maternal pro-inflammatory cytokines—interleukin-6 (IL-6), interleukin-1β (IL-1β), and tumor necrosis factor-α (TNF-α)—traverse the placental barrier, directly activate fetal microglia, and polarize these cells toward an M1 pro-inflammatory phenotype, thereby initiating a neuroinflammatory cascade.

At the molecular level, IL-6 engages membrane-bound IL-6 receptor (mIL-6R) and triggers Janus kinase-signal transducer and activator of transcription (JAK-STAT) signaling, down-regulating paired-box protein 6 (PAX6) and promoting premature differentiation of radial glial cells (RGCs). This process disrupts neural stem cell (NSC) self-renewal and impairs neurogenesis [171, 172]. IL-1β binds IL-1 receptor type 1 (IL-1R1), activates nuclear factor-κB (NF-κB), and stimulates microglial generation of reactive oxygen species (ROS) and TNF-α, damaging neural progenitor cells while simultaneously activating the NOD-like receptor family pyrin domain containing 3 (NLRP3) inflammasome, thus establishing a positive-feedback loop that amplifies inflammation [173]. IL-17A phosphorylates Claudin-5, disrupting BBB integrity and promoting peripheral immune cell infiltration [174].

These inflammatory insults collectively suppress NSC proliferation, disrupt neuronal migration and cortical lamination, impair synaptogenesis, and shift excitation–inhibition balance while reducing hippocampal excitability, culminating in learning, memory, and emotional deficits that mirror core NDD behavioral phenotypes in animal models [175].

Prospective cohorts show that maternal immune dysregulation, chronic inflammation, and trans-placental autoantibodies alter fetal brain trajectories by disrupting neurogenesis, synaptic plasticity, and neurotransmitter balance [176]. Future work must map spatiotemporal actions of individual mediators across critical windows and develop targeted immunomodulation to enable early risk detection and primary prevention of NDDs.

## Neuroimmune interactions in peripheral disorders

Neuroimmune interactions are integral to both central nervous system (CNS) pathology and a spectrum of peripheral disorders. Emerging evidence delineates their mechanistic contributions to gut–brain-axis regulation, chronic and neuropathic pain, and oncogenesis as well as responses to anti-cancer therapy.

### The gut-brain axis

**Fig. 3:**
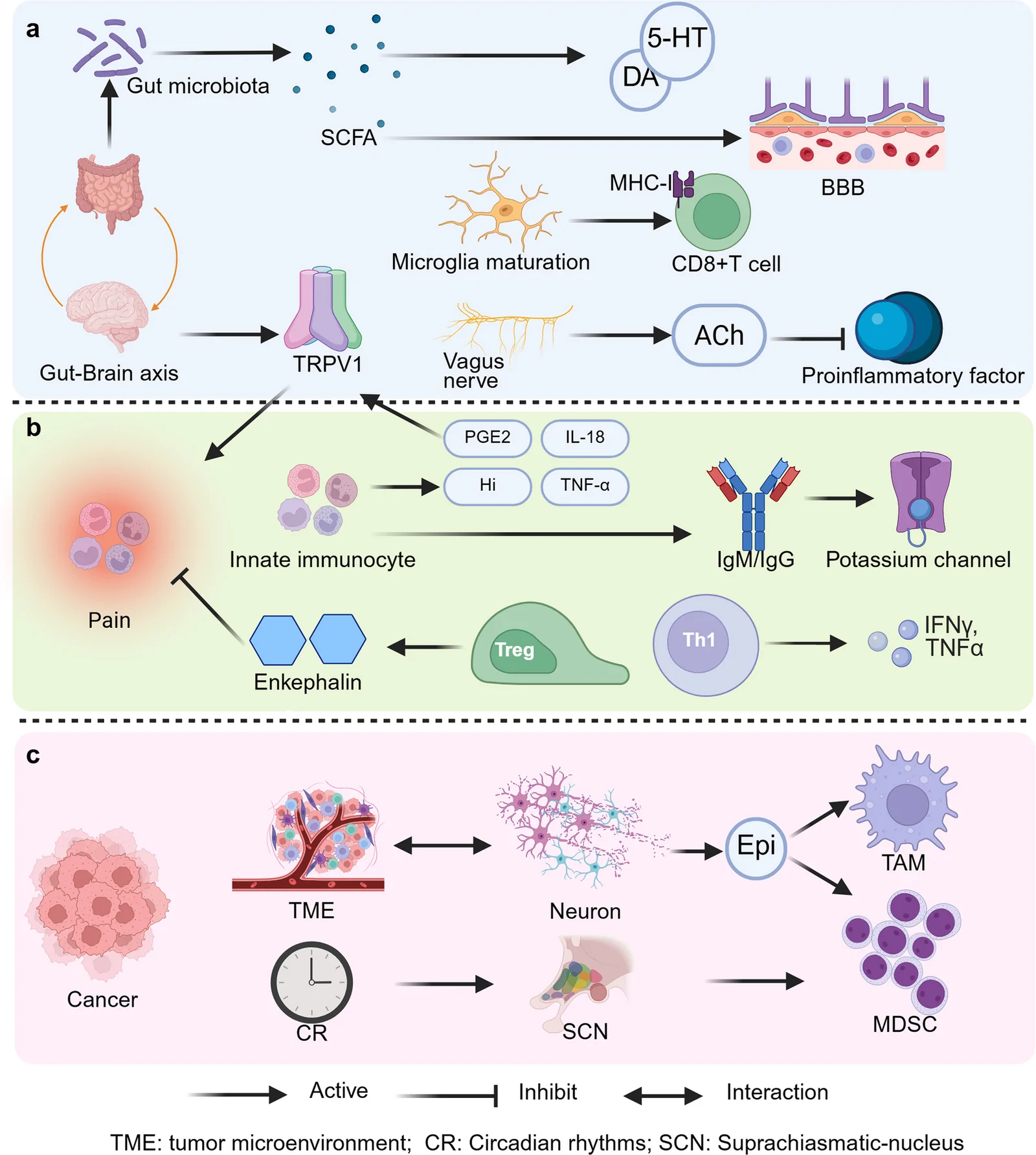
Neuroimmune interactions in the gut–brain axis, pain processing, and the tumor microenvironment. **a** In the gut-brain axis, the gut microbiota regulates neuroimmune communication through short-chain fatty acid (SCFA) production, induction of regulatory T cells (Tregs), and vagal signaling, thereby influencing microglial maturation and central immune tolerance. **b** During pain processing, innate immune cells promote hyperalgesia by releasing cytokines and antibodies that modulate ion channel activity, whereas Tregs exert analgesic effects through interleukin-10 (IL-10) and enkephalin secretion. **c** In the tumor microenvironment, neural tissue and circadian rhythms bidirectionally regulate tumor-associated immune responses, including the activation of myeloid-derived suppressor cells (MDSCs). (Created with BioRender.com. BioRender agreement number: BKNBL36Y5M)

The gut–brain axis constitutes a bidirectional neuroanatomical and biochemical communication network in which intestinal microbiota modulate brain function and behavior through metabolic, immune, and neuroendocrine pathways. Dysregulation of this axis contributes to the pathogenesis of multiple neurodegenerative and psychiatric disorders (Fig. 3).

#### Microbiome-immune-neuro communication

The gut microbiota constitutes a core regulatory node of the gut–brain axis, modulating central nervous system (CNS) physiology through metabolic, immune, and neuroanatomical pathways. Dysbiosis disrupts these pathways and precipitates neuropsychiatric pathology. Microbiota-derived signals are transmitted via three principal mechanisms: (1) Metabolic: bacterial fermentation of dietary fibre yields short-chain fatty acids (SCFAs) that promote microglial maturation and function [177], and commensals synthesize neurotransmitters including serotonin and dopamine; (2) Immune: colonization-induced differentiation of regulatory T cells (Tregs) maintains CNS immune tolerance, whereas dysbiosis provokes chronic low-grade inflammation that accelerates neurodegeneration and psychiatric symptomatology [178]; (3) Neural: microbial metabolites and cell-wall components signal through the vagus nerve to influence affective and cognitive processing. Perturbation of this bidirectional dialogue impairs neurotransmitter homeostasis, synaptic plasticity, and adult neurogenesis [179].

Germ-free or broad-spectrum antibiotic-treated mice exhibit an immature microglial transcriptomic and morphological profile; defective microglial maturation is rescued by recolonization with defined SCFA-producing taxa or by direct SCFA supplementation [180]. Lipopolysaccharide (LPS) exerts concentration-dependent, polarity-specific effects, driving microglia toward either a neuroprotective or pro-inflammatory phenotype [181]. Tryptophan-derived microbial metabolites activate the aryl hydrocarbon receptor (AHR) in microglia and astrocytes, thereby inhibiting nuclear factor-κB (NF-κB) signaling and attenuating neuroinflammation [182]. Additionally, the microbiota educates meningeal lymphocyte populations and preserves BBB integrity; germ-free mice display increased BBB permeability secondary to reduced tight-junction protein expression, and reconstitution with SCFA-producing bacterial strains restores barrier function [183].

Concurrent systemic and CNS immune dysregulation characterises neurodevelopmental, psychiatric and neurodegenerative disorders, prompting clinical evaluation of targeted microbiota interventions (diet, next-generation probiotics, screened faecal transplantation); yet microbiota–brain causality remains unclear, heterogeneity is high, and efficacy plus long-term safety await validation in adequately powered randomised trials.

#### Neuroimmune mechanisms in Inflammatory Bowel Disease (IBD)

Inflammatory bowel disease (IBD) is propelled by a genetically primed immune attack on commensal microbiota whose course—whether Crohn’s or ulcerative colitis—is orchestrated by bidirectional enteric–central neuroimmune crosstalk [184]. Hyper-excitable extrinsic afferents release substance P/CGRP to recruit mast cells and effector T cells [185], while enteric glia up-regulate MHC-I and activate CD8⁺ T cells—enhancing antimicrobial defence yet amplifying neuronal apoptosis and barrier loss [186, 187]. Stress-driven HPA activation elevates glucocorticoids/catecholamines that skew mucosal immunity toward inflammation and increase permeability; conversely, sustained colitis remodels limbic–prefrontal circuits, heightening depression/anxiety risk and closing a pathological gut–brain loop [188]. Microbiota-derived butyrate/propionate and tryptophan–indole metabolites induce Treg differentiation, tighten epithelial junctions and activate microglial/astrocytic aryl-hydrocarbon receptor signaling to preserve BBB integrity; dysbiosis depletes these metabolites, precipitating barrier leakage and meningeal immune dysfunction. TRPV1 mediates visceral hyperalgesia, CGRP exerts context-dependent anti-inflammatory effects and TRPM8 activation is protective [189], while IL-17⁺ Th17 cells expand on pathobiont challenge and ZEB2 up-regulation cripples Treg suppression. Macrophages adopt region-restricted phenotypes—pro-inflammatory lamina-propria subsets versus muscularis populations under sympathetic neuromodulation [190]. Targeted β3-adrenergic agonists, TRPV1 antagonists, precision probiotics and rigorously screened faecal microbiota transplantation are therefore being evaluated to restore immune tolerance, reseal the epithelial barrier and recalibrate gut–brain homeostasis in IBD.

#### The vagus nerve as a key modulator

The vagus nerve—cranial nerve X—serves as the principal parasympathetic conduit of the neuroimmune axis, relaying real-time visceral information to the nucleus tractus solitarii and directing rapid cerebral modulation of peripheral immunity [191, 192]. Its efferent arm, the cholinergic anti-inflammatory pathway, releases acetylcholine that engages α7-nicotinic receptors on macrophages, triggering Ca^2^⁺-dependent blockade of NF-κB and silencing TNF-α, IL-1β and IL-6 [193]. Vagus-nerve stimulation—approved for refractory epilepsy and depression—now shows promise in Crohn’s disease, rheumatoid arthritis and chronic pelvic pain by not only suppressing cytokines but also driving lipoxin A₄/resolvin D₁ biosynthesis and autophagic clearance of cellular debris to accelerate resolution [194, 195]. These findings establish the vagus as a tractable neuromodulatory target and underscore the neuroimmune axis as a central regulator of homeostasis.

### Chronic pain and neuropathic pain

Chronic pain is now viewed as a neuroimmune disorder [196]. Peripheral sensitisation begins when mast-cell, macrophage- and T-cell-derived mediators lower nociceptor thresholds, producing hyperalgesia and allodynia; centrally, microglia propagate spinal and supraspinal sensitisation via IL-1β, IL-18, TNF-α and ROS-dependent synaptic potentiation [197, 198]. Reciprocal glia–neuron signaling sustains this loop [199], so microglial modulators, cytokine antagonists or vagal neuromodulation offer disease-modifying analgesia.

#### Peripheral sensitization and immune-cell infiltration

Peripheral sensitisation is initiated and maintained by a spatially and temporally organised neuro–immune circuit at the site of tissue or nerve injury. Damaged cells, severed axons, and the first wave of infiltrating neutrophils, macrophages, and mast cells generate an “inflammatory soup” rich in prostaglandin E₂ (PGE₂), interleukin-1β (IL-1β), histamine, and tumor necrosis factor-α (TNF-α) [200]. These mediators directly gate nociceptor sodium channels (Nav1.7, Nav1.8) or sensitize transient-receptor-potential vanilloid-1 (TRPV1) receptors, thereby lowering activation thresholds and producing stimulus-independent action-potential discharge [201]. Autoantibodies secreted by locally recruited B cells amplify the process. In rheumatoid arthritis, anti-citrullinated-protein antibodies (ACPA) activate macrophages via Fc-γ receptors and complement C5a signaling [202] whereas in complex regional pain syndrome IgM/IgG autoantibodies bind voltage-gated potassium channels (Kv1.1, Kv1.2) and impair neuronal repolarisation, accelerating the acute-to-chronic transition [203]. Neuropeptides released from nociceptors (substance P, CGRP) further dilate post-capillary venules and recruit additional immune cells, establishing a self-sustaining neurogenic inflammatory loop. T lymphocytes exert bidirectional control at this stage: interferon-γ (IFN-γ)-producing Th1 cells enhance macrophage activation and nociceptor sensitisation, whereas regulatory T cells (Treg) release IL-10 and met-enkephalin to initiate endogenous analgesic programmes [204, 205]. Targeted blockade of TNF-α, IL-1β, or complement C5a, and adoptive transfer of antigen-specific Treg, are therefore rational, mechanism-based strategies to interrupt the vicious cycle and provide disease-modifying relief from chronic pain.

#### Central sensitization and glial activation

Central sensitization denotes a maladaptive increase in nociceptive network excitability that sustains chronic pain [206]. The process is actively orchestrated by spinal and supraspinal glia together with dynamic infiltration of peripheral immune cells. Microglia—CNS-resident macrophages—are first responders to nerve injury or inflammation: they release brain-derived neurotrophic factor (BDNF) that collapses chloride homeostasis via down-regulation of KCC2, and secrete IL-1β and TNF-α that enhance presynaptic transmitter release and postsynaptic N-methyl-D-aspartate receptor (NMDAR) currents, thereby engraving a “pain memory” [207]. Astrocytes concurrently lose their capacity to buffer extracellular K⁺ and glutamate, and produce chemokines CXCL1 and CXCL13 that recruit immune cells and consolidate hyperexcitability [208]. Sustained nociceptive input increases blood–spinal-cord and BBB permeability, enabling infiltration of neutrophils, CD4⁺ and CD8⁺ T cells, and B-cell-derived autoantibodies. T-cell-derived interferon-γ (IFN-γ) amplifies astrocytic reactivity, whereas autoantibodies characteristic of complex regional pain syndrome type I (anti-CRPS-I IgM) activate microglia through complement C5a–C5aR1 signaling, establishing a self-reinforcing neuroimmune loop [209]. This bidirectional glial–immune drive not only produces aberrant synaptic plasticity and hyperalgesia but also precipitates affective comorbidities and opioid tolerance [210]. Consequently, pharmacological or biological blockade of microglial activation (CSF1R inhibitors), C5a–C5aR1 signaling, or IFN-γ–IFNGR1 interactions constitutes a mechanistically grounded strategy to interrupt central sensitisation and achieve disease-modifying analgesia.

Despite these advances, substantial challenges persist. Neuroinflammation displays marked spatiotemporal heterogeneity across pain phenotypes, complicating target selection, and systemic immunomodulation carries infection, cardiovascular, and neoplastic risks that require rigorous evaluation in adequately powered trials.

### Cancer and cancer therapy

The neuroimmune–tumor axis—the tripartite crosstalk among neurons, immune cells, and cancer cells—governs tumor initiation, progression, metastasis, and therapeutic resistance [211]. Axonogenic sprouting from autonomic and sensory ganglia infiltrates the tumor microenvironment (TME), where neurons secrete neurotransmitters (e.g., noradrenaline, acetylcholine, glutamate) and neuropeptides (e.g., substance P, CGRP) that directly activate oncogenic signaling cascades in malignant cells and reprogram immune effectors toward a pro-tumoural phenotype [212]. Reciprocally, immune-derived cytokines and growth factors stimulate neuronal hyper-excitability and neurogenesis, establishing a self-amplifying feedback loop. This bidirectional communication is not epiphenomenal but an active driver of tumorigenesis. Therapeutic exploitation of the axis is twofold: (i) pharmacological or surgical denervation suppresses tumor growth and potentiates checkpoint blockade, and (ii) cholinergic or adrenergic receptor antagonists restore cytotoxic T-cell function and reverse myeloid-mediated immunosuppression [211]. Single-cell RNA sequencing and spatial transcriptomics have now resolved neuron-like cancer-cell subpopulations and mapped their physical proximity to regulatory T cells and exhausted CD8⁺ T cells, providing high-resolution targets for precision intervention [213].

#### The role of nerves in the tumor microenvironment

The tumor microenvironment (TME) is an actively orchestrated, immunosuppressive ecosystem in which the peripheral nervous system constitutes a third pillar of tumor immune evasion. Sympathetic norepinephrine (NE) engages β2-adrenergic receptors (β2-AR) on myeloid-derived suppressor cells (MDSCs) and tumor-associated macrophages (TAMs), enhancing fatty-acid oxidation, up-regulating PD-L1 and arginase-1, and driving M2 polarization while simultaneously suppressing dendritic-cell (DC) maturation and type-I interferon responses [214, 215]. Sensory nerves release calcitonin gene-related peptide (CGRP) that impairs DC antigen presentation and induces CD8⁺ T-cell exhaustion via RAMP1 [216], conversely, substance P (SP) amplifies macrophage cytokine secretion yet promotes thyroid-cancer-cell survival, illustrating context-dependent neuroimmune modulation [217]. Parasympathetic acetylcholine activates α7-nicotinic receptors on TAMs, triggering TNF-α secretion and promoting memory T-cell-derived TFF2 that restrains MDSC expansion [218]. Schwann-cell-derived CCL2 recruits immunosuppressive TAMs, and their Toll-like receptors detect tumor-derived DAMPs to further attenuate immune surveillance [219]. Neuroendocrine cells sense hypoxia and release serotonin or histamine, epigenetically up-regulating tumor PD-L1 [220]. These peripheral neural programmes are systemically coordinated by brain–autonomic reflex arcs (e.g., insula–sympathetic axis) [221], forming a neuroimmune symbiotic network that fuels tumor proliferation, invasion, and metastasis.

#### Neuroimmune interactions in cancer progression and metastasis

The neuroimmune axis fosters tumor progression by generating a microenvironment permissive for invasion and dissemination [222]. Chronic stress activates sympathetic nerves; norepinephrine stimulates β-adrenergic receptors on macrophages, up-regulates metastatic gene signatures and triggers neutrophil extracellular traps that ensheath circulating tumor cells. Perineural invasion exemplifies local co-operation: tumor-derived NGF and artemin evoke nociceptive sprouting and tumor–nerve synapses whose CGRP/substance-P output accelerates proliferation, migration and angiogenesis [223]. In pancreatic ductal adenocarcinoma, hypoxia- and lactate-conditioned tumor-associated macrophages (TAMs) secrete CCL5, recruiting CCR5-expressing carcinoma cells to the perineural niche and establishing a self-reinforcing loop that simultaneously drives PNI and neuropathic pain [224, 225]. Neuroimmune signals display context-dependent, dichotomous roles. In glioma, visual-experience-evoked neuronal activity induces CD8⁺ T cells to release midkine-dependent CCL4, which stimulates microglial CCL5 production and fuels tumor growth—an example of CD8⁺ T cells exerting tumor-promoting functions. Conversely, CGRP⁺ sensory afferents suppress CD8⁺ T-cell surveillance in melanoma, illustrating immune inhibition by nociceptive signaling. Systemic metastatic propensity is amplified by circadian and gut–brain axis disruption. Suprachiasmatic-nucleus (SCN) dysfunction dampens macrophage BMAL1 expression, stabilises hypoxia-inducible factor-1α (HIF-1α) and skews macrophages toward an M2 phenotype, while clock-gene mutations expand myeloid-derived suppressor cells (MDSCs) and up-regulate PD-L1 [226]. Dysbiosis reduces microbial butyrate production, compromises blood–brain barrier (BBB) integrity and enables meningeal macrophages to secrete glial-cell-line-derived neurotrophic factor (GDNF), thereby facilitating breast-cancer-cell meningeal colonization [227]. Collectively, neural signals act as systemic coordinators that couple local invasion to distant metastasis by reprogramming immune-cell metabolism and phenotypic plasticity, constructing a metastasis-supportive neuroimmune network.

#### Impact of cancer therapies on the neuroimmune axis

The neuroimmune axis functions as a bidirectional rheostat that both constrains and amplifies the efficacy of contemporary immuno-oncotherapies. Sustained adrenergic signalling ligates β2-adrenergic receptors (β2-AR) on myeloid and lymphoid compartments, expanding myeloid-derived suppressor cells (MDSCs), polarising tumor-associated macrophages (TAMs) toward an M2 phenotype, and impairing dendritic-cell (DC) maturation, thereby establishing an “immune brake” that limits immune-checkpoint inhibitor (ICI) efficacy [228]. Conversely, non-selective β-blockade with propranolol disrupts this cascade: in randomised trials, propranolol plus anti-PD-1 increases intratumoural CD8⁺ T-cell density, decreases PD-1 expression on cytotoxic T lymphocytes, and improves circulating metastatic biomarkers in early-stage breast cancer and melanoma [229]. ICI-induced clinical benefit is, however, counter-balanced by neurological immune-related adverse events (nirAEs) such as Guillain–Barré syndrome, and by off-target neurotoxicity of cytotoxic chemotherapy or radiotherapy that activates damage-associated molecular pattern (DAMP)–Toll-like receptor (TLR) pathways, promoting aberrant nerve sprouting and intractable neuropathic pain [230]. Consequently, broader neuroimmune modulation strategies are under investigation. CGRP-neutralising monoclonal antibodies reverse sensory-nerve-mediated CD8⁺ T-cell exhaustion; RORγ agonists re-educate TAM metabolism to potentiate chimeric antigen receptor (CAR) T-cell function; and faecal microbiota transplantation restores gut–brain axis homeostasis and augments ICI responsiveness [231, 232]. Collectively, these data underpin a precision-oncology framework stratifying patients by tumor innervation, β-AR expression, and stress indices, with TSC22D3 guiding the timing of neuroimmune modulators; concurrent optogenetic tuning of the VTA–prefrontal circuit promises real-time calibration of the neuro–immune–tumor triad to maximise anti-tumor immunity while minimising neurotoxicity.

## Molecular mechanism-based therapeutic strategies and targets

Molecular mechanisms provide the mechanistic bridge between neuroimmune communication and disease pathology. At the cellular level, microglia, astrocytes, endothelial cells, peripheral leukocytes, and neurons respond to danger signals and inflammatory mediators through interconnected receptor–ligand networks. At the intracellular level, TLR/NLR signaling, the NLRP3 inflammasome, NF-κB, MAPK, JAK–STAT, cGAS–STING, complement, and oxidative-stress pathways regulate cytokine production, cellular metabolism, barrier integrity, synaptic remodeling, and cell survival. These molecular circuits determine whether neuroimmune interactions remain protective or become pathogenic. Advances in the understanding of neuroimmune communication have expanded the therapeutic landscape beyond conventional immunosuppression toward mechanism-based intervention. Rather than broadly suppressing inflammation, current strategies aim to selectively modulate disease-driving pathways while preserving protective immune functions. As discussed above, these approaches can be broadly grouped into four categories: precision biologics, small-molecule inhibitors and repurposed agents, cell-based and neuromodulatory therapies, and microbiome-directed interventions. Their clinical utility depends on disease context, dominant pathogenic mechanism, and the extent to which central or peripheral neuroimmune circuits are involved.

### Precision biologics

Precision biologics represent the most direct way to neutralize defined cytokines, chemokines, complement components, or neuropeptides that drive neuroimmune pathology. In MS, blocking inflammatory mediators such as GM-CSF, CCL2/CCR2, or VLA-4 can reduce peripheral immune-cell trafficking into the CNS and dampen downstream glial activation [233–235]. In neurodegenerative disorders, biologic inhibition of complement activation and cytokine signaling may limit synaptic pruning, microglial overactivation, and neuronal injury [236]. Similar principles apply in AE, NMOSD, and pain disorders, where antibody-mediated or complement-driven mechanisms are central to pathology [95–97, 137]. In oncology, biologics targeting CGRP or immune checkpoint-associated neuroimmune pathways are being explored to reverse nerve-mediated immunosuppression and improve antitumor immunity [216, 228]. The chief advantages of this approach are specificity and pathway selectivity; however, limitations include incomplete blood–brain barrier penetration, infection risk, high cost, and the need for careful patient stratification based on molecular phenotype [237, 238, 239].

### Small-molecule inhibitors and repurposed agents

Small-molecule inhibitors provide a second therapeutic tier by targeting intracellular signaling nodes that integrate multiple upstream inflammatory inputs. Inhibition of the NLRP3 inflammasome, MAPK pathways, IDO signaling, or oxidative-stress cascades can attenuate cytokine amplification and reduce glial or peripheral immune-cell activation across several disorders [15, 16, 103, 115, 148, 155, 240]. For example, MCC950 has shown promise in experimental autoimmune encephalomyelitis, while CCR2 antagonists can reduce leukocyte recruitment in models of CNS inflammation and pain [234, 235]. In schizophrenia and depression, antioxidant and ferroptosis-related interventions may be relevant where oxidative injury and immune dysregulation coexist [152–156]. Repurposed drugs expand this category further: β-blockers may counter adrenergic stress signaling in cancer and inflammatory disease, whereas GLP-1 receptor agonists and other metabolic modulators are being investigated for neurodegenerative conditions [237, 241]. These agents are attractive because they are often easier to deploy clinically than novel biologics, but their therapeutic window can be narrow, and off-target effects remain a major challenge (Table 2).

**Table 2:** Key Molecular pathways and druggable targets in the neuroimmune axis

Pathway/molecule	Physiological role	Disease-specific dysregulation	Therapeutic agent (Example)	Target diseases
NLRP3 Inflammasome	Innate immune activation by DAMPs/PAMPs	Overactivated by Aβ, α-syn, mSOD1; drives IL-1β release [103, 115, 240]	MCC950	AD, PD, ALS, MS
CCL2/CCR2 Axis	Monocyte/T-cell chemotaxis	Mediates peripheral infiltration across BBB in AD, pain, MS [107, 237]	CCR2 antagonists (PF-4136309)	AD, chronic pain, MS
Complement (C1q/C3/C5a)	Synaptic pruning, immune clearance	Aberrant synapse elimination in AD, NMOSD; MAC formation [131]	Eculizumab (anti-C5a)	NMOSD [44]; trials in AD
Adrenergic β2-AR	Stress response, immune modulation	Promotes MDSC/TAM immunosuppression in cancer & neurodegeneration [210, 224, 225]	Propranolol (non-selective β-blocker)	Cancer (adjunct) [134]; PD (observational)
CGRP/Substance P	Sensory neurotransmission	Sensory nerve-mediated T-cell exhaustion, tumor promotion [211]	CGRP mAbs (erenumab)	Cancer (preclinical)
IDO/Kynurenine	Tryptophan metabolism, immunoregulation	IL-6-driven IDO↑ → 5-HT↓, neurotoxic metabolites in depression [143, 144]	IDO1 inhibitors (navoximod)	Depression (adjunct)
GM-CSF	Myeloid cell differentiation & activation	GM-CSF⁺ Th cells drive CNS inflammation in MS, neurodegeneration [25, 26]	Anti-GM-CSF mAb (mavrilimumab)	MS, ALS
AHR Signaling	Xenobiotic/immune modulation	Microbiota-derived agonists↓ → NF-κB↑ in neuroinflammation [178]	AHR agonists (ITE)	ASD, MS
MMP8	ECM remodeling	Stress-induced release modifies reward-circuit synapses [145]	MMP8i (selective inhibitors)	Depression
Glutamate/Excitotoxicity	Synaptic transmission	Astrocyte uptake↓ → motor neuron death in ALS, PD	NMDA antagonists (memantine)	ALS (failed), PD (investigational)

↑: Increase; →: Active; ↓: Decline

### Cell-based and neuromodulatory therapies

Cell-based and neuromodulatory interventions aim to restore immune balance at the level of cell populations or neural circuits rather than suppressing individual inflammatory mediators. Regulatory T-cell transfer, mesenchymal stromal cells, and engineered CAR-T platforms are being developed to re-establish immune tolerance or eliminate pathogenic immune subsets in CNS autoimmune disease, cancer, and chronic inflammatory states [231–233, 242, 243]. In parallel, neuromodulation approaches such as vagus nerve stimulation, transcutaneous auricular stimulation, and repetitive transcranial magnetic stimulation can engage endogenous anti-inflammatory pathways and reshape microglial or macrophage phenotypes [191–195, 242]. These approaches are especially relevant to disorders in which bidirectional neuroimmune loops, rather than a single dominant cytokine, sustain disease progression, as described in Sects. "Neuroimmune interactions in central nervous system disorders" and "Neuroimmune interactions in peripheral disorders" for pain, depression, and cancer. Their main advantages are systems-level reprogramming and the possibility of closed-loop control; nevertheless, challenges include delivery, durability, parameter optimization, and procedural complexity.

### Microbiome-directed interventions

Microbiome-directed therapies target the gut-brain axis, which influences CNS function through metabolic, immune, and neural routes. By restoring short-chain fatty acid production, tryptophan metabolism, and barrier integrity, these interventions may reduce peripheral inflammation and normalize microglial maturation and T-cell homeostasis [177–180]. In disorders such as MS, autism spectrum disorder, depression, inflammatory bowel disease, and PD, dysbiosis is increasingly recognized as a contributor to neuroimmune imbalance, making dietary intervention, next-generation probiotics, prebiotics, and fecal microbiota transplantation attractive adjunctive strategies [164, 165, 178, 179, 188, 227]. These approaches are conceptually well aligned with the peripheral-to-central signaling pathways discussed earlier, particularly the microbiota-immune-neural axis and vagus nerve-dependent communication. However, heterogeneity in donor composition, colonization stability, and patient responsiveness currently limits reproducibility and clinical translation (Table 3).

**Table 3:** Emerging therapeutic modalities: mechanisms, status, and challenges

Modality	Clinically approved/advanced	Preclinical/exploratory	Target disease (s)
Biologics: mAbs/Cytokines	Anti-CGRP (erenumab), anti-IL-6R (tocilizumab), eculizumab		Migraine, NMOSD, depression (adjunct)
Small-Molecule Inhibitors	propranolol (β-blocker), IDO1i	MCC950 (NLRP3i),	AD, PD, cancer, pain
Cell-Based Therapies	MSCs, CAR-T cells	co-transplantation of regulatory T cells (Tregs)	MS, ALS, glioblastoma
Neuromodulation	rTMS, VNS	optogenetics, BCI	Depression, IBD, chronic pain
Microbiome Interventions	FMT, Lactobacillus probiotics	SCFAs	ASD, depression, PD
Nanoparticle Delivery	Liposomes, exosomes	polymer NPs	AD, glioblastoma, pain
Gene Silencing/Editing	–	siRNA (TREM2, NLRP3), CRISPR (microglia)	AD, ALS (familial)
Complement Inhibitors	–	C1q-blocking peptide, C3aR antagonist	AD, NMOSD
Metabolic Reprogramming	α7-nAChR agonist	PERK-ATF4 inhibitors (ISRIB)	GBM, stroke, MS
Repurposed Drugs	β-blockers, GLP-1 agonists, thalidomide	Metformin, DPP4 inhibitors	Cancer, AD, neuropathic pain

Overall, these four therapeutic avenues reflect a shift toward personalized neuroimmune medicine. Future progress will depend on biomarker-guided patient stratification, combination strategies, and interventions that match the dominant pathogenic layer—whether molecular, cellular, circuit-level, or microbiome-related—while minimizing systemic toxicity.

## Conclusion

This review establishes neuroimmune interactions as a fundamental principle governing CNS homeostasis and pathology across diverse disorders, demonstrating that traditional boundaries between neuroscience and immunology are obsolete. We must embrace a unified framework of integrated networks with profound implications for disease understanding and therapy development.

In conclusion, to move beyond a purely integrative synthesis of existing frameworks, we propose a mechanistically grounded classification of neuroimmune disorders based on the predominant immune effector pathway. In this framework, neuroimmune diseases can be broadly stratified into antibody-driven, T cell-driven, and glia-driven disorders, while acknowledging that many conditions likely involve overlapping mechanisms and may evolve along this spectrum over time. This classification is intended as a conceptual tool to better account for clinical heterogeneity, pathobiological diversity, and variable therapeutic responsiveness across neuroimmune syndromes, and it further generates a testable hypothesis that the dominant immune mechanism is a major determinant of clinical phenotype, disease course, and treatment response. Accordingly, humoral-dominant diseases may preferentially respond to B cell- or complement-targeted therapies, whereas T cell-dominant or glia-dominant diseases may be more amenable to interventions targeting lymphocyte trafficking, cytokine signaling, or glial reprogramming. Key outstanding questions remain: how to distinguish protective from pathogenic immune responses across disease stages, whether distinct immune endotypes can be reliably identified using multimodal biomarkers, how age, sex, genetic background, and the microbiome shape immune predominance, and how single-cell, spatial and longitudinal multi-omics data can be integrated into clinically actionable models. Addressing these questions will be essential for translating neuroimmune biology into a more precise, mechanism-based framework for diagnosis and therapy.

## Data Availability

No data was used for the research described in the article.
